# Rictor—A Mediator of Progression and Metastasis in Lung Cancer

**DOI:** 10.3390/cancers16030543

**Published:** 2024-01-26

**Authors:** Fatime Szalai, Dániel Sztankovics, Ildikó Krencz, Dorottya Moldvai, Judit Pápay, Anna Sebestyén, Andras Khoor

**Affiliations:** 1 Department of Pathology and Experimental Cancer Research, Semmelweis University, Üllői út 26, H-1085 Budapest, Hungary; fatie.salay@gmail.com (F.S.); sztankovics.daniel@gmail.com (D.S.); krencz.ildiko@gmail.com (I.K.); moldvai.dorottya@gmail.com (D.M.); papayjudith@gmail.com (J.P.); hsebanna@gmail.com (A.S.); 2 Department of Laboratory Medicine and Pathology, Mayo Clinic, 4500 San Pablo Road, Jacksonville, FL 32224, USA

**Keywords:** lung cancer, mTOR pathway, mTORC2, *RICTOR* amplification, Rictor overexpression

## Abstract

**Simple Summary:**

Despite recent advances in targeted therapy and immunotherapy, the treatment of lung cancer remains challenging due to its high metastatic potential, which adversely affects both the prognosis and the quality of life of these patients. Rictor, the scaffold protein of the mTORC2 complex, plays an important role in regulating essential cellular functions and promoting metastases through epithelial–mesenchymal transition. Amplification of the *RICTOR* gene and subsequent overexpression of the Rictor protein can activate mTORC2 and promote cell survival and migration. Recent studies suggest that *RICTOR* amplification and Rictor overexpression may serve as markers of mTORC2 activation. Potentially, they can also provide druggable targets for advanced therapy. Although these drugs are still in a preclinical phase, selective mTORC2 inhibitors are a promising approach to inhibit tumor cell migration and metastasis formation. This review highlights the importance of Rictor and mTORC2 as predictive markers and promising therapeutic targets in the treatment of lung cancer.

**Abstract:**

Lung carcinoma is one of the most common cancer types for both men and women. Despite recent breakthroughs in targeted therapy and immunotherapy, it is characterized by a high metastatic rate, which can significantly affect quality of life and prognosis. Rictor (encoded by the *RICTOR* gene) is known as a scaffold protein for the multiprotein complex mTORC2. Among its diverse roles in regulating essential cellular functions, mTORC2 also facilitates epithelial–mesenchymal transition and metastasis formation. Amplification of the *RICTOR* gene and subsequent overexpression of the Rictor protein can result in the activation of mTORC2, which promotes cell survival and migration. Based on recent studies, *RICTOR* amplification or Rictor overexpression can serve as a marker for mTORC2 activation, which in turn provides a promising druggable target. Although selective inhibitors of Rictor and the Rictor-mTOR association are only in a preclinical phase, they seem to be potent novel approaches to reduce tumor cell migration and metastasis formation. Here, we summarize recent advances that support an important role for Rictor and mTORC2 as potential therapeutic targets in the treatment of lung cancer. This is a traditional (narrative) review based on Pubmed and Google Scholar searches for the following keywords: Rictor, *RICTOR* amplification, mTORC2, Rictor complexes, lung cancer, metastasis, progression, mTOR inhibitors.

## 1. Introduction—Lung Cancer and mTOR

Based on the GLOBOCAN 2020 estimates of cancer incidence and mortality, lung cancer is the second most common malignancy worldwide, with about 2.2 million new cases (11.4% of all cancer cases in 2020) [1]. Despite recent breakthroughs in targeted and immunotherapy, it remains the leading cause of cancer-related death (18% of all cancers) [1]. This mortality can be partly explained by the high metastatic potential of the disease, and the fact that most patients already have metastatic disease at diagnosis. At the time of diagnosis, 75% of tumors cannot be removed surgically, leaving systemic therapy and radiotherapy as the only treatment options for these patients [2,3]. These findings underscore the urgent need for new, effective therapeutic options that may also have an impact on the metastatic propensity of the tumor, which is an important determinant of the prognosis [4,5].

The most common histological subtypes of lung cancer are adenocarcinoma (ADC), squamous cell carcinoma (SCC), and small cell lung carcinoma (SCLC). From a clinical point of view, ADCs, SCCs, and LCCs comprise a group referred to as non-small cell lung cancer (NSCLC), accounting for 80–85% of all lung carcinomas. Small cell lung cancer (SCLC), although less common (15% of all cases), has a particularly aggressive clinical behavior; most patients present with distant metastases at the time of diagnosis [6,7]. While a wide range of treatment options (including targeted and immunotherapy or even surgical resection in the early stage) is available for NSCLCs, the therapeutic armamentarium for SCLC is more limited [8].

**Figure 1:**
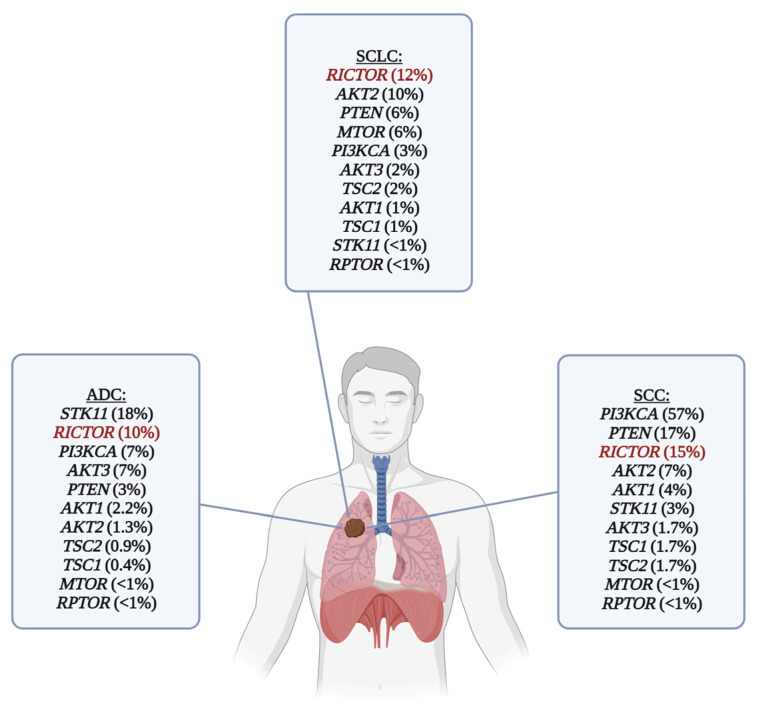
Frequency of genetic alterations of the mTOR pathway in the most common histological subtypes of lung cancer. *RICTOR* amplification is the most common targetable genetic alteration in SCLC, and it is also one of the most frequent genetic alterations in lung ADC and SCC. *RICTOR* is highlighted with red color. For ADC and SCC, data (mutations and copy number alterations) were obtained from The Cancer Genom Atlas (Firehose Legacy) via www.cbioportal.org (downloaded on 20 December 2023). Only the alterations marked as putative drivers are shown in the picture. SCLC data were obtained from various studies [9,11,12,13,14,15], a subset of which [11,12,13,14] was also downloaded from www.cbioportal.org (downloaded on 20 December 2023). List of abbreviations: ADC, adenocarcinoma; SCC, squamous cell carcinoma; SCLC, small cell lung carcinoma.

Many molecular pathomechanisms of lung cancer have been identified over the last two decades. Aside from the well-known genetic alterations, such as *EGFR* and *KRAS* mutations or *ALK* and *ROS1* rearrangements in lung adenocarcinoma, mutations or copy number variations of the phosphatidylinositol 3-kinase/protein kinase B/mammalian target of rapamycin (PI3K/Akt/mTOR) pathway members also frequently occur in lung cancer and may also serve as therapeutic targets [9,10]. The most common genetic alterations of the mTOR pathway in lung cancer are shown in Figure 1.

The mechanistic/mammalian target of rapamycin (mTOR) is a serine/threonine kinase known as a master regulator of cellular metabolism. It is involved in integrating environmental signals (hormones, growth factors, oxygen, etc.) and the subsequent activation or inhibition of essential cellular pathways related to proliferation, growth, and survival. In the absence of adequate molecular signals or energy sources, it can also initiate cell survival mechanisms, such as autophagy, at the expense of cell growth [16]. The mTOR kinase is tightly linked to the PTEN/PI3K/Akt axis and other essential molecular pathways (e.g., LKB1-AMPK pathway), creating an extensive and complex signaling network [17,18]. Because of its diverse role in regulating essential cellular functions, alterations in mTOR signaling can lead to the development of a wide range of diseases, including metabolic, cardiovascular, or neurodegenerative disorders, accelerated aging, and cancer [19,20].

The mTOR kinase can be a part of two structurally and functionally distinct multiprotein complexes: mTOR complex 1 (mTORC1) and mTOR complex 2 (mTORC2). Both complexes share the common subunits mammalian lethal with SEC13 protein 8 (mLST8) and DEP domain-containing mTOR-interacting protein (DEPTOR). Specific protein subunits of mTORC1 are regulatory-associated proteins of mTOR (Raptor), acting as a scaffold protein and proline-rich Akt substrate of 40 kDa (PRAS40). mTORC2 has a rapamycin-insensitive companion of mTOR (Rictor) instead of Raptor, a mammalian stress-activated protein kinase-interacting protein 1 (mSin1), and a protein observed with Rictor 1 and 2 (Protor 1/2) [21]. Having Rictor as a scaffold unit creates a unique spatial structure for mTORC2, as the FKBP-rapamycin binding site is placed inside the molecule, rendering the mTORC2 inaccessible to the conventional mTOR inhibitor drug, rapamycin (also known as sirolimus) [22].

**Figure 2:**
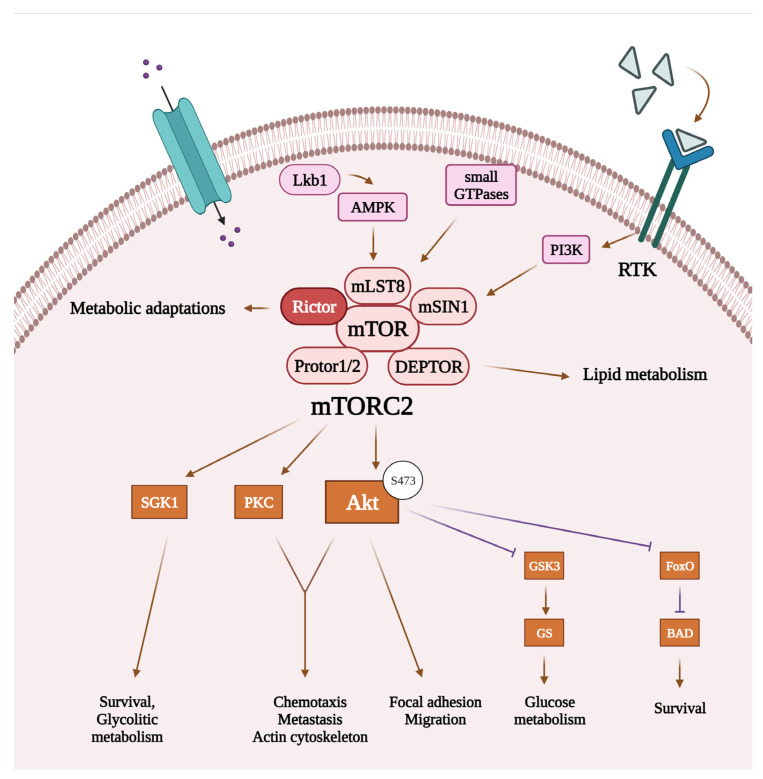
Cellular functions regulated by mTORC2 and related signaling pathways. Recent studies suggest that mTORC2 can be activated by receptor tyrosine kinases through PI3K signaling, AMP-activated protein kinase, or small GTPases. Once activated, mTORC2’s main targets include Akt, SGK 1, and PKC. By activating its targets, mTORC2 can regulate bioenergetic processes and actin cytoskeleton reorganization, thereby promoting survival and migration of the tumor cells. List of abbreviations: Akt, protein kinase B; AMPK, AMP-activated protein kinase; BAD, BCL2 associated agonist of cell death; DEPTOR, domain-containing mTOR-interacting protein; FoxO, forkhead box protein O1; GS, glycogen synthase; GSK3, glycogen synthase kinase-3; mLST8, mammalian lethal with SEC13 protein 8; mSin1, mammalian stress-activated map kinase-interacting protein 1; mTOR, mammalian target of rapamycin; mTORC2, mTOR complex 2; PI3K, phosphatidylinositol 3-kinase; PKC, protein kinase C; Protor1/2, protein observed with Rictor 1/2; Rictor, rapamycin-insensitive companion of mTOR; RTK, receptor tyrosine kinase; SGK1, serum glucocorticoid-regulated kinase 1.

While it is well-known that mTORC1 enables anabolic processes such as protein, lipid, and nucleotide synthesis or ribosome biogenesis and controls cellular metabolism, mTORC2 is also involved in the regulation of a wide range of cellular functions, including bioenergetic processes (see Figure 2) [23]. Although the upstream regulation of mTORC2 remains less well characterized compared to mTORC1, there is emerging evidence that several kinases, such as receptor tyrosine kinases, AMP-activated protein kinases, or small GTPases can act on mTORC2 or its scaffold protein Rictor to regulate the activation of mTORC2 [9,19,23]. Upon activation, mTORC2 can phosphorylate its downstream targets, which include Akt, serum glucocorticoid-regulated kinase 1, and protein kinase C. Of these, Akt protein seems to be one of the most important targets, which, upon phosphorylation (at Ser473), affects the expression of oncogenic transcription factors [17,23]. It has also been described that mTORC2 plays a crucial role in cell migration by regulating the actin cytoskeletal system and epithelial–mesenchymal transition (EMT), which are important aspects of tumor cell invasion and metastasis formation [23,24,25,26,27]. In various types of cancer, mTORC2 and Rictor have been implicated in the regulation of the tumor microenvironment through angiogenesis or stromal remodeling, interacting with vascular endothelial growth factor A, one of the key molecules in these processes [28].

Hyperactivation of the mTOR complexes resulting from the genetic alterations of the members of the PI3K/Akt/mTOR signaling cascade is frequently seen in solid tumors, including the most common histological subtypes of lung cancer [9,22,29]. The prevalence of mTOR pathway alterations was 50–70% in NSCLCs (90% of ADCs, 40% of SCCs, and 60% of LCCs) and 36% in SCLCs [9,22,30]. Mutational hotspots for amino acid changes in the *MTOR* gene, encoding for the mTOR kinase, are 1799K, T1977R, V2006F, S2215Y, and R2505P, which could reduce the binding of DEPTOR, the inhibitory subunit of the mTOR complexes, and thus increase the phosphorylation and activation of target molecules, such as p70 S6 kinase and eukaryotic translation initiation factor 4E binding protein 1 [31,32]. Structural changes in the mTOR complexes can also negatively affect the binding to allosteric mTOR inhibitors (such as F2108L mutation of the *MTOR* gene in the FKBP–rapamycin binding (FRB) domain), leading to therapy resistance [33]. In addition to mutations affecting the mTOR kinase, impaired signaling can occur through genetic alterations involving various other genes encoding for proteins implicated in the PI3K/Akt/mTOR pathway (e.g., *PIK3CA*, *PTEN*, *AKT1*, *AKT2*, *AKT3*, *TSC1*, *TSC2*, *STK11*) [23] or affecting one of the subunits of the mTOR complexes (e.g., *RICTOR*) (see Figure 1) [34].

Identifying the specific molecular changes in the background of mTOR hyperactivation has undeniable clinical benefits, as these alterations could serve as therapeutic targets for selected patients [9,35]. As new therapeutic options have emerged, the need to characterize mTOR activity in malignant tumors has increased, particularly in cancers with elevated mTORC2 activity, which may not respond to traditional mTOR inhibitors that only target mTORC1 [36]. Evaluation of mTOR activity could be achieved by assessing the active phosphorylated form of mTOR (p-mTOR) or its phosphorylated target molecules (e.g., p-4EBP1, p-p70S6K, or p-S6) using immunohistochemistry (IHC). For mTORC2 activity, recent studies suggest that analysis of Rictor and p-Akt (Ser473) expression is the most effective method used to predict the response to mTORC1/2 inhibitors. The mTORC1/mTORC2 ratio can also be assessed by evaluating the ratio of their respective scaffold subunits, Raptor and Rictor, respectively. In summary, the optimal in situ marker combination for assessing mTOR activity in tumors may include p-mTOR, Raptor, and Rictor, as well as an additional target protein of mTORC1 (such as p-S6, p-70S6K, p-4EBP1) and mTORC2 (p-Akt (Ser473)) [37,38]. However, it is important to note that the detection of phosphorylated proteins in formalin-fixed paraffin-embedded sections can be difficult and unreliable in some cases due to the inconsistent sample processing, especially in clinical samples. For example, rapid and proper sample processing is particularly important for the detection of p-Akt (Ser473) protein because of the potential for rapid dephosphorylation [39].

## 2. Rictor-mTOR Association—The mTORC2 Protein Complex and Its Functions

Rictor (encoded by the *RICTOR* gene) was identified as a novel component of mTORC2 in 2004 [40]. As a scaffolding subunit, its primary function is to guide the assembly of mTORC2 and maintain its structural integrity [17,20]. One of the well-known oncogenic alterations of the mTOR pathway is the *RICTOR* amplification, which has been shown to contribute to progression and metastasis in certain cancers through its association with the molecular signaling behind these processes (e.g., Wnt/β-katenin, MAPK/ERK pathways) [41]. Several studies have demonstrated both *RICTOR* amplification and Rictor overexpression in different types of cancer, including lung cancer [9,17,42,43,44,45,46,47]. Moreover, both *RICTOR* amplification and Rictor overexpression have been identified to be associated with poor prognosis and shorter survival [17,28,48]. In addition to amplification, a few other mechanisms (e.g., glucose-dependent acetylation or DNA methylation) have also been described to regulate Rictor expression and, therefore, mTORC2 activation [48,49]. However, further investigations are needed to reveal the exact role of these processes.

As previously mentioned, mTORC2 has an important role in the regulation of cell survival and organization of the actin cytoskeleton system. Moreover, besides the extensive roles of the LKB1/AMPK signaling and mTORC1 in the regulation of bioenergetic processes [50,51], mTORC2 has also been implicated in metabolic processes: its activation has been described to be enhanced by amino acid (especially glutamine) withdrawal or glucose starvation [52]. Recent studies have also elucidated that, besides mTORC1, mTORC2 also plays essential functions in the maintenance of metabolic homeostasis [53].

Emerging evidence suggests that the kinase activity of mTORC2 may also depend on the subcellular localization. Recruitment of mTORC2 to the plasma membrane plays an important role in its activation and enables the phosphorylation of one of its major downstream targets, Akt, which is also recruited to the plasma membrane through its pleckstrin homology domain [54]. In addition to its distribution along the plasma membrane, it has also been detected in the cytosol and the nuclei. In the cytosol, mTORC2 can be related to various subcellular structures, such as mitochondria, endosomes, lysosomes, Golgi complex, and endoplasmic reticulum [55,56]. A recent study on glioblastoma cells has suggested that mTORC2 translocates from the plasma membrane to the nuclear and perinuclear compartments when mTORC2 is inactivated [57]. However, there is still controversy over whether mTORC2 can translocate among different subcellular compartments or whether its location is static. The latter is in correlation with the findings that there are distinct forms of mTORC2, which are defined by specific isoforms of the components in the complex, particularly mSin1, and sensitivity to PI3K signaling [58]. This topic has become increasingly complex over the years, and the exact role of subcellular localization in the regulation and functions of mTORC2 remains to be elucidated.

## 3. Other Rictor-Containing Protein Complexes

Recent findings suggest that, in addition to mTORC2, Rictor may be part of other protein complexes with potential oncogenic or tumor-suppressing properties, further expanding its important role in tumorigenesis. One of the most studied partner proteins is the integrin-linked kinase (ILK), which interacts with β1-integrin. The Rictor/ILK complex has been reported to play an essential role in transforming growth factor beta-1 (TGFβ1)-mediated EMT in vitro, and the complex has also been shown to phosphorylate Akt on Ser473 in an mTOR-independent manner [59]. Other Rictor partners include Culin-1: its complex gains E3 ubiquitin-ligase activity and contributes to the degradation of the mTORC2 effector serum/glucocorticoid-induced kinase 1 [60]. Another Rictor-containing complex in human glioma cells consists of tetraspanin 8 and integrin a3. This complex was found to be required for the assembly and proper function of mTORC2 in glioblastoma cells [61]. Rictor can also interact with the myosin Myo1c and activate paxillin to influence cortical actin remodeling [62]. The co-localization of Rictor and protein kinase Cζ has also been reported, and their complex plays a pivotal role in cancer chemotaxis and metastasis formation in breast cancer [63]. The role of Rictor/F-box WD repeat containing 7 complexes with tumor suppressor properties has also been described in colorectal cancer cells, targeting the oncogenes c-Myc and cyclin E [64]. Moreover, in renal cancer, Rictor can interact with programmed cell death protein 4 at the expense of mTORC2, thereby reducing the metastatic ability of the tumor cells [65]. Although increasing evidence supports the essential role of the Rictor/ILK complex in the regulation of EMT [66,67,68], further studies are still needed to elucidate the clinical significance of these protein complexes. Taken together, it is hypothesized that Rictor acts as a universal scaffold protein and, therefore, can exert different biological functions independent of the mTORC2 complex [17,69].

## 4. The Role of Rictor in the Epithelial–Mesenchymal Transition and Migration of the Tumor Cells

**Figure 3:**
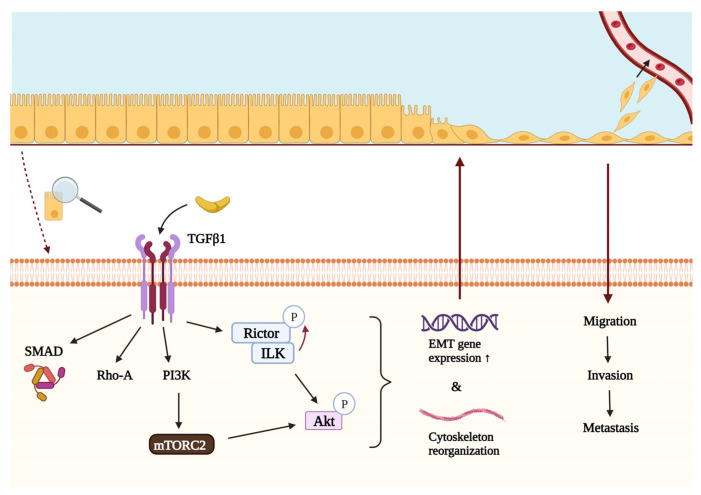
The role of Rictor in the TGFβ-mediated epithelial–mesenchymal transition and metastasis formation. TGFβ1 is an important regulator of epithelial–mesenchymal transition and thus metastasis formation. In addition to Rho-A and Smad proteins, it can interact with PI3K, which activates mTORC2 and leads to the subsequent phosphorylation of Akt on Ser473 and initiation of downstream effector processes. Akt can also be phosphorylated by another protein complex containing Rictor and ILK. The activity of ILK is also required for the phosphorylation of Rictor in Thr1135, which can regulate the assembly of mTORC2. Once activated by either mTORC2 or the ILK/Rictor complex, Akt regulates cellular functions that can promote epithelial–mesenchymal transition and metastasis formation. List of abbreviations: Akt, protein kinase B; EMT, epithelial–mesenchymal transition; ILK, integrin-linked kinase; mTORC2, mTOR complex 2; PI3K, phosphatidylinositol 3-kinase; Rho-A, Ras homolog family member A; Rictor, rapamycin-insensitive companion of mTOR; SMAD, suppressor of mothers against decapentaplegic; TGFβ1, transforming growth factor β1.

EMT is a process by which epithelial cells undergo a phenotypic change to become motile mesenchymal cells capable of invasion. For this change to occur, cells have to lose their junctions (prominently E-cadherin) and apical–basal polarity, reorganize the cytoskeleton, and reprogram gene expression and signaling related to cell shape and motility [25]. Mesenchymal–epithelial transition (MET) describes the reverse process in which cells regain their epithelial properties. EMT occurs in physiological conditions such as embryogenesis and wound healing, but it has also been shown to play a crucial role in cancer progression and metastasis formation [69,70]. EMT promotes the dissociation, migration, and invasion of tumor cells, enabling them to metastasize to distant organs, where they revert to their original epithelial nature during MET. Since EMT is known to promote invasiveness and metastatic activity of the tumor, it is strongly correlated with a worse prognosis [71,72]. Several reports suggest that Rictor may play a central role in these processes (see Figure 3) [59,69,73,74,75].

One of the most important mechanisms regulated by mTORC2 is cell migration via reorganization of the actin cytoskeleton, which may contribute to metastasis formation [76]. Recent studies on neutrophil migration have revealed that membrane tension can act on actin nucleation and polymerization to regulate protrusion formation. Increasing membrane tension activates a mechanosensitive signaling cascade and stimulates mTORC2, which controls a negative feedback mechanism in maintaining the homeostasis of membrane stretch [77,78]. It has also been shown in glioblastoma cells that *RICTOR* knockdown and subsequent inactivation of mTORC2 significantly decrease tumor cell migration by affecting the actin cytoskeleton and microtubule organization [57]. Thus, by integrating divergent cytoskeletal programs, mTORC2 is an essential regulator of cell polarity and migration and, therefore, metastasis formation.

TGFβ1 is a key regulator of growth, differentiation, and epithelial transformation in tumorigenesis, interacting with PI3K, Rho-A, and Smad proteins [25,59]. Another fundamental molecule during TGFβ1-induced EMT is the aforementioned ILK, which connects the cytoplasmic domains of β-integrins to the actin cytoskeleton. When interacting with Rictor, the ILK/Rictor complex can phosphorylate Akt on Ser473, initiating downstream effector processes [66]. It has been described that TGFβ1 can activate mTORC2 to enhance Rictor expression and ILK/Rictor complex formation in an ILK-dependent manner, thereby increasing Akt activation. ILK can also phosphorylate Rictor on Thr1135, a site that is also responsive to growth factor stimuli, PI3K activity, and ribosomal protein S6 kinase 1 activity, which is a downstream effector of mTORC1 (Figure 3). In vitro knockdown of ILK and Rictor suppresses TGFβ1-induced EMT in tumor cells, highlighting the fundamental role of the ILK/Rictor complex in mediating this process. It was also found that the ILK/Rictor complex forms in cancer cells but not in normal cells, making it a promising target for cancer-specific therapy that is not harmful to normal cells [17,59].

Increased ribosome biogenesis regulated by the mTORC2 is also an important factor of EMT. In the G1/S cell cycle arrest during EMT, Rictor has been found to allocate to the nucleoli to associate with the newly formed ribosomes. The mTORC2 complex can also bind to ribosomes, contributing to mesenchymal gene expression [79]. In summary, Rictor is a crucial effector of TGFβ1-induced EMT not only through mTORC2 activation but also individually and as part of the Rictor/ILK complex [59].

## 5. *RICTOR* Amplification and/or Rictor Overexpression in Lung Cancer

Identification of *RICTOR* amplification serves as a predictive marker for effective inhibition of the PI3K/mTOR/Akt pathway, particularly increased mTORC2 activity. The gold standard method for detecting *RICTOR* amplification is fluorescence in situ hybridization (FISH). Moreover, next-generation sequencing (NGS) or Droplet Digital PCR (ddPCR) can detect the *RICTOR* copy number variations. In situ, Rictor expression can be assessed by IHC or immunocytochemistry, depending on the sample type [38,80].

*RICTOR* amplification has been found in 10% of lung ADCs, 16% of lung SCCs, and 23.6% of lung neuroendocrine tumors [17]. In lung ADCs, Rictor overexpression has been described in 37% of the primary cases [37]. *RICTOR* amplification has been identified as one of the most common targetable genetic alterations in SCLC, with a prevalence ranging from 6 to 15% [9,81]. Moreover, a positive correlation between the presence of *RICTOR* amplification and Rictor expression has been described in multiple studies [28,38,46]. In some cases, Rictor overexpression has been found at a higher rate than *RICTOR* amplification, which can be explained by other genetic alterations in the mTOR pathway, epigenetic changes, or miRNA expression aberrations in the tumor cells [82,83].

In the clinical setting, patients with *RICTOR*-amplified SCLC are expected to have shorter overall survival [81]. Similar results have been found in a study investigating the expression of mTOR pathway markers in lung ADC: Rictor protein expression has been assessed in primary and brain metastatic lung ADCs; Rictor and Rictor/mTOR levels have been increased in the brain metastases, and elevated Rictor expression has also been associated with a higher stage of the primary tumor [17,37]. Similar trends have been observed in SCLC, as Rictor expression has been found to be significantly higher in brain metastases compared to primary tumors and lymph node metastases [38]. In contrast to the above-mentioned study by Sakre et al. [81], this study could not confirm the association between the presence of *RICTOR* amplification and unfavorable clinical outcome. However, the high expression of both Rictor and p-Akt was associated with shorter overall survival [38]. Moreover, p-Akt and Rictor could also serve as surrogate markers to identify lung cancer patients with an increased probability of responding to double mTORC1/2 inhibitors [47].

These findings further highlight the importance of detecting *RICTOR* amplification or Rictor overexpression in lung cancer, as they may serve as markers of aggressive clinical behavior and therapy sensitivity, especially in cases in which *RICTOR* amplification is the only targetable oncogenic driver [38].

## 6. Selective Inhibitors of mTORC2—Targeting the Association of Rictor and mTOR

**Figure 4:**
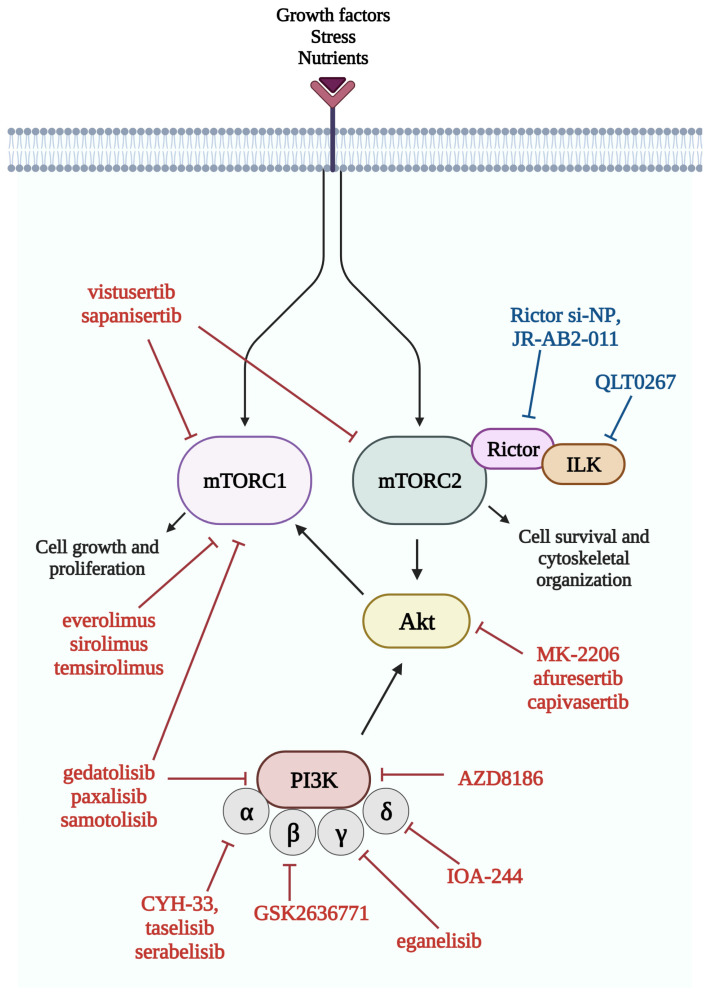
Members of the mTOR pathway and their inhibitors. Several inhibitors of the mTOR pathway have been developed in recent years, and most of them are currently studied in clinical trials. Red color indicates inhibitors, which are currently studied in clinical trials, while blue color indicates inhibitors in the preclinical phase. List of abbreviations: Akt, protein kinase B; ILK, integrin-linked kinase; mTORC1, mTOR complex 1; mTORC2, mTOR complex 2; PI3K, phosphatidylinositol 3-kinase; Rictor, rapamycin-insensitive companion of mTOR.

Over the past decade, a large number of PI3K/Akt/mTOR pathway inhibitors have been developed (Figure 4), but despite the many ongoing clinical trials, the number of approved drugs remains low. Inadequate patient selection due to a lack of predictive markers may explain this phenomenon. By studying certain genetic alterations (loss-of-function mutation of *PTEN*, *AKT* mutation, and *RICTOR* amplification) or the overexpression of the activated pathway molecules (such as p-S6 or p-Akt), the increased efficacy of mTOR inhibitors could be anticipated [84].

The most well-known mTOR inhibitor, rapamycin, was originally isolated as an antibiotic and was only later recognized as an immunosuppressant and anti-tumoral agent when a transplant patient with kidney cancer received rapamycin treatment [85,86]. Following the discovery of its target, the mTOR complex, rapamycin (or sirolimus), and its analogs (everolimus, temsirolimus) became therapeutic options for patients with various types of cancer (e.g., breast, kidney, or central nervous system tumors), but clinical results have been highly variable. Moreover, the effect of most mTOR inhibitors in monotherapy is lacking [9,22,84,87,88]. In regard to mTORC1 inhibitors, this phenomenon can be explained by the existence of feedback loop mechanisms; inhibition of the mTORC1/S6K1-mediated negative feedback paradoxically increases PI3K and mTORC2 activity, leading to phosphorylation of Akt [89]. As described, rapamycin binds mTOR through its FKBP domain, which is unavailable in the mTORC2 complex due to structural dissimilarity, so the effect of rapamycin analogs in targeting mTORC2 hyperactivated tumors warrants further investigation [90].

Double kinase inhibitors inhibiting the activity of both mTORC1 and mTORC2 (e.g., CC-115, sapanisertib, vistusertib) and dual kinase inhibitors inhibiting the activity of mTOR kinase and other members of the signaling pathway (e.g., PI3K, such as edatolisib, paxalisib, samatolisib) are under investigation. Akt inhibitors (afuresertib, capivasertib, ipatasertib, TAS-117, triciribin, uprosertib, and in lung cancer: MK-2206) are also being studied in phase II and III trials for the treatment of advanced cancers (NCT01147211). In lung cancer specifically, gedatolisib (a dual inhibitor of PI3K and mTOR) is being tested in a phase I trial in combination with the CDK4/6 inhibitor palbociclib in NSCLC (NCT03065062). mTOR inhibitors combined with immune checkpoint inhibitors are also being evaluated in NSCLC (NCT04348292). The MEK inhibitor selumetinib is being studied in both NSCLC and SCLC in combination with vistusertib and temsirolimus (NCT02583542). In a phase I study, the combination of vistusertib and paclitaxel has been found to increase clinical response in SCC [91].

Specific targeting of mTORC2 while preserving mTORC1 activity remains a pharmacological challenge, although drugs based on siRNA technology (Rictor si-NP) or small molecule inhibitors targeting the mTOR-Rictor interaction (JR-AB2-011) are being investigated in the preclinical setting [92,93]. mTOR inhibitors and their combination with other targeted therapies currently under clinical investigation in lung cancer are shown in Table 1.

Among lung neoplasms, sporadic lymphangioleiomyomatosis (LAM) is the only entity for which an mTOR inhibitor is approved as a first-line treatment option [94]. The FDA approved sirolimus for the treatment of LAM in 2015, which has served as the cornerstone of therapy ever since. However, it has also been noted that not all subgroups of LAM patients respond well to conventional mTOR inhibition; moreover, intolerable side effects can lead to treatment discontinuation [95,96]. Thus, there is an urgent need to explore alternative therapeutic options for this disease, as well. In addition to mTORC1, evidence for increased mTORC2 activity has also been found in LAM cells, suggesting a potential clinical benefit from the use of dual kinase inhibitors [97]. A study investigating vistusertib in animal models has shown that it inhibits EMT and tumor progression in tuberous sclerosis-associated tumors; however, clinical data on the efficacy of vistusertib in these tumors are not yet available [98].

Despite the clear association between hyperactivation of the PI3K/Akt/mTOR pathway and poor prognosis in many tumor types, including lung cancer, the efficacy of mTOR inhibitors as monotherapy or in combinations with other targeted therapies is unsatisfactory. Furthermore, frequent and sometimes severe side effects, which require careful management by clinicians, continue to hinder its widespread and efficient use [36]. However, the combination of mTOR inhibitors with chemotherapy or radiotherapy can counteract the development of resistance mechanisms and lower the incidence of unwanted side effects. New therapeutic regimens are currently being investigated to increase the efficacy of mTOR inhibitors in inhibiting tumor progression and improving survival [87].

As the understanding of genetic changes leading to the development of lung cancer increases and as predictive markers are being discovered, there is an increasing trend in clinical trials that patients are selected based on the molecular characteristics of the tumor. As an example, vistusertib monotherapy has been tested in specifically selected patients with SCLC who have a confirmed *RICTOR* amplification. In this study, four patients have been declared eligible, two of whom had their survival extended by almost one year following vistusertib administration [99]. These findings represent a promising avenue for developing novel therapies and individualized treatment [100].

## 7. Conclusions and Future Perspectives

Despite recent breakthroughs in targeted and immunotherapy of lung cancer, the prognosis for most patients remains poor [1]. Metastasis formation is one of the major determinants of survival and can also significantly affect life quality of the patients [2]. As a part of mTORC2, Rictor has been shown to be an important regulator of EMT and migration of the cancer cells by regulating the actin cytoskeleton, which in turn can facilitate the metastatic ability of the tumor [24,25].

Inhibition of mTORC2 seems to be a promising target in preventing or decreasing metastasis formation [93]. However, emerging results on different Rictor-containing complexes and the role of subcellular localization in the regulation of mTORC2 [55,59] can influence and further complicate the results in both preclinical and clinical studies and still need to be elucidated.

Inhibitors targeting the mTOR kinase, thereby inhibiting the activity of both mTORC1 and mTORC2, are currently studied in clinical trials for the treatment of different histological types of lung cancer (Table 1), but the results are variable. The lack of biomarker-based patient selection could be one of the main reasons behind the inefficacy of these inhibitors in previous studies. However, the start of several biomarker-based clinical trials in recent years is promising and may lead to the clinical translation of these drugs. Selective inhibitors of mTORC2 are also available in preclinical trials with encouraging results.

In conclusion, despite the increasing number of various inhibitors of the mTOR pathway tested in clinical trials, they have not yet achieved a breakthrough in the treatment of lung cancer. Identifying predictive biomarkers may improve the clinical translation of these inhibitors. To predict the response to mTORC2 or mTORC1/C2 inhibitors, *RICTOR* amplification and overexpression of Rictor and p-Akt are currently considered the best markers [80]. A recent study analyzing *RICTOR* amplification by next-generation sequencing, droplet digital PCR, and fluorescence in situ hybridization, as well as Rictor and p-Akt expression by immunohistochemistry on a large set of tumors of various origins has demonstrated the superiority of the traditional fluorescence in situ hybridization in the detection of *RICTOR* amplification. However, other molecular methods as well as Rictor and p-Akt immunohistochemistry can be used to select patients for further analysis [38,80]. Analyzing the mTORC2 activity of the tumor may also predict the metastatic potential of the cancer cells and thus the prognosis, thereby guiding treatment decisions.

## Figures and Tables

**Table 1:** Selected clinical trials evaluating inhibitors of the PI3k/Akt/mTOR pathway in lung cancer.

Class	Drug(s)	Target Tumor Type	Biomarker-Based Selection?	Phase	Identifier
PI3K inhibitors	alpelisib	NSCLC	Yes	II	NCT04591431
	GSK2636771	Lung cancer	*PTEN* mutation or deletion	II	NCT02465060
	idelalisib + pembrolizumab	NSCLC	No	I/II	NCT03257722
	IPI-549 + nivolumab	NSCLC	No	I	NCT02637531
	serabelisib + canagliflozin	Lung cancer	*PIK3CA* mutation, *KRAS* mutation	I/II	NCT04073680
	taselisib	Lung cancer	*PIK3CA* mutation without *KRAS* mutation or *PTEN* loss	II	NCT02465060
Akt inhibitors	capivasertib	NSCLC	Yes	II	NCT02664935
	capivasertib	NSCLC	Yes	II	NCT02117167
	capivasertib	Lung cancer	*AKT* mutation	II	NCT02465060
	ipatasertib	Lung cancer	*AKT* mutation	II	NCT02465060
	ipatasertib + atezolizumab	NSCLC	No	I/II	NCT03337698
	ipatasertib + docetaxel	NSCLC	No	II	NCT04467801
	MK-2206 + gefitinib	NSCLC	No	I	NCT01147211
mTORC1 inhibitors	everolimus + ceritinib	NSCLC	Yes	I	NCT02321501
	everolimus	NSCLC	Yes	II	NCT04591431
	everolimus	Lung neuroendocrine carcinoma	No	II	NCT02687958
	everolimus + VS-6766	Lung cancer	Yes	I	NCT02407509
	everolimus + trametinib + lenvatinib	Lung cancer	No	II	NCT04803318
	nab-sirolimus + adagrasib	NSCLC	Yes	I/II	NCT05840510
	sirolimus + epacadostat	NSCLC	No	I	NCT03217669
	temsirolimus + solumetinib	Lung cancer	No	I	NCT00600496
	RAPA-201	Lung cancer	No	I/II	NCT05144698
Double mTORC1/2 inhibitors	sapanisertib + osimertinib	NSCLC	Yes	I	NCT02503722
	vistusertib + durvalumab	NSCLC	Biomarker-matched and biomarker-non-matched cohorts	II	NCT03334617
	vistusertib + selumetinib	NSCLC	*KRAS* mutation	I/II	NCT02583542
	vistusertib	NSCLC	Yes	II	NCT02664935
	vistusertib	NSCLC	Yes	II	NCT02117167
	vistusertib	SCLC	Yes	II	NCT03106155
Dual PI3K and mTORC1/2 inhibitors	gedatolisib + palbociclib	SCC	Yes	I	NCT03065062

Data were downloaded from www.ClinicalTrials.gov on 10 November 2023. List of abbreviations: Akt, protein kinase B; KRAS, Kirsten rat sarcoma virus; mTORC1, mTOR complex 1; mTORC1/2, mTOR complex 1 and 2; NSCLC, non-small cell lung cancer; PI3K, phosphatidylinositol 3-kinase; PIK3CA, phosphatidylinositol 3-kinase catalytic subunit alpha; PTEN, phosphatase and tensin homolog deleted in chromosome 10; SCC, squamous cell carcinoma; SCLC, small cell lung carcinoma.

## Data Availability

A publicly available dataset was analyzed in this study, The Cancer Genom Atlas (Firehose Legacy), via www.cbioportal.org (downloaded on 20 December 2023).
